# Weibull Statistical Analysis of Strength Fluctuation for Failure Prediction and Structural Durability of Friction Stir Welded Al–Cu Dissimilar Joints Correlated to Metallurgical Bonded Characteristics

**DOI:** 10.3390/ma12020205

**Published:** 2019-01-09

**Authors:** Chung-Wei Yang, Shiau-Jiun Jiang

**Affiliations:** Department of Materials Science and Engineering, National Formosa University, No. 64, Wunhua Road, Huwei, Yunlin 63201, Taiwan; 10152114@gm.nfu.edu.tw

**Keywords:** friction stir welding, dissimilar joints, metallurgical bonding microstructure, the Weibull model, failure strength, engineering reliability

## Abstract

In this paper, dissimilar Al–Cu joints of AA1050H/C1100-Cu, AA6061-T6/C1100-Cu, and AA1050H/C2600-brass are successfully welded by a friction stir welding (FSW) process. The aim of the present study is not only to examine the tensile strength, but also to investigate the reliability, durability, and failure behaviors of joints as correlated with the metallurgical bonded microstructures of varied Al–Cu joints. Experimental evidence confirms that good welding quality for an FSW Al–Cu dissimilar joint is obtained when pure Cu and brass plates are positioned at the advancing side. Cross-sectional microstructures reveal that the AA6061-T6/C1100-Cu joint exhibits an extensive metallurgical bonded region with significant onion rings in the welding zone, whereas the AA1050H/C2600-brass joint generally displays a clear mechanical kissing bonded boundary at the joint interface. Al_2_Cu, Al_4_Cu_9_, and γ-Cu_5_Zn_8_ are major intermetallic compounds (IMCs) that are formed within the metallurgical bonded welding zone. The Weibull model provides a statistical method for assessing the failure mechanism of FSW Al–Cu joints. Better welding reliability and tensile properties with ductile dimpled ruptures are obtained for the Al–Cu joints with a typical metallurgical bonded zone. However, a mechanical kissing bonded interface and thick interfacial IMCs result in the deterioration of tensile strength with a brittle fracture and a rapid increase in the failure probability of Al–Cu joints.

## 1. Introduction

The welding of dissimilar metals is becoming an important subject for industrial applications nowadays due to their technical and beneficial advantages [1,2,3,4,5]. Aluminum (Al) and copper (Cu) are two common engineering metals with favorable mechanical strength, ductility, and good corrosion resistance. Moreover, Al–Cu dissimilar joints have been widely used in engineering structural components, electronic packaging, and the electric power industries, and are of interest in electrical connections because of their excellent electrical and thermal conductivities. In contrast with these advantages, the joining of dissimilar Al and Cu alloys is generally difficult by a conventional fusion welding process due to the differences in their physical, thermal, and mechanical characteristics. Thus, the development of a promising welding technique for joining dissimilar Al and Cu alloys has been made by a number of researches [6,7,8,9,10].

Friction stir welding (FSW) is a solid-state joining process [11,12] that was first invented by The Welding Institute (TWI) of the United Kingdom (UK) [13], and can be considered an important development in joining dissimilar metals [11]. The FSW process is considered energy efficient and environmentally friendly because no toxic fumes are produced during the welding process. FSW can be commonly used to join nonferrous light and plastic metals such as Al, Mg and Ti alloys with other dissimilar metal alloys that are hard to weld by conventional fusion welding [2,5,14,15,16,17,18]. Recently, FSW has also been recognized as an effective technique to overcome the welding problems of Al–Cu dissimilar joints [19,20,21,22,23,24,25,26,27]. Some studies indicated that not only the welding quality, but also mechanical properties of Al–Cu dissimilar joints are significantly influenced by controlling the FSW parameters [21,22,23,24,25] and microstructural features, especially for the intermetallic compounds (IMCs) layer formed at the bonding interface within the welding zone of Al–Cu dissimilar joints [26,27,28,29,30,31]. The brittleness of the IMCs layer usually results in easier cracks propagation and failures at the joint interface [32,33]. However, the enhanced mechanical properties of Al–Cu joints can also be achieved by controlling the particle size and distribution of the IMCs within the welding zone of FSW joints [26,28,32].

Since the failure of FSW-joined structural components depends on the applied stress to approach a critical weakest strength within the welding zone, the variability of the failure strength and the durability of FSW joints are fairly correlated with the welding qualities. Therefore, it is worthwhile to investigate the correlation of microstructural features to the data fluctuation of mechanical strength and welding reliability of FSW dissimilar joints in detail. The failure prediction can be effectively achieved through a statistical reliability engineering method [34], and the Weibull model [34,35] of survival analysis has been developed as a popular and powerful engineering design method for various structural materials and joint performance [36,37,38,39,40]. The advantage of Weibull statistical analysis is that it provides reasonably accurate failure analysis and failure predictions with a small number of samples. Solutions can be acquired at earlier indications of problems, and fewer samples also enable cost-effective component testing.

Therefore, in order to clarify the influence of metallurgical factors on the failure strength and the durability of FSW Al–Cu dissimilar joints, the aim of the present study is to examine the microstructural features of the welding zone and evaluatethe mechanical strength of dissimilar joints under tensile tests. In addition, a statistical analysis of the Weibull model with the examination of fracture surface morphologies will be applied to investigate the welding reliability, joints durability, and failure mechanism of FSW Al–Cu dissimilar joints.

## 2. Materials and Methods

The base metals that were used in this study were three-mm thick commercial pure copper (99.9% purity, annealed, JIS C1100), brass (JIS C2600), AA1050H, and AA6061-T6 aluminum rolled sheets. The chemical compositions of raw materials that are given in Table 1 were determined by inductively coupled plasma-atomic emission spectrometry (ICP-AES). The base metals were machined into rectangular specimens with dimensions of 80 (*l*) × 30 (*w*) × 3 (*t*) mm for the FSW process. The surfaces of FSW-joined specimens were ground with 2000-grit SiC papers, and then ultrasonically cleaned with acetone prior to welding. The friction stir welded AA1050H/C1100, AA6061-T6/C1100, and AA1050H/C2600 dissimilar joints were carried out in this study, and these joints were labeled as A1/C1, A6/C1, and A1/C2 specimens in the following, respectively.

**Figure 1:**
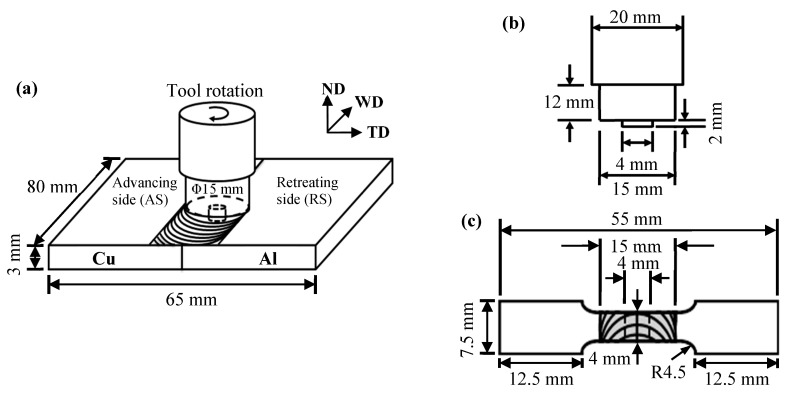
Schematic illustrations of the (**a**) friction stir welding (FSW) process of Al–Cu dissimilar joints, (**b**) the welding tool geometries used for FSW, and (**c**) the dimensions of the tensile specimen prepared from welded samples.

Figure 1a schematically illustrates the FSW process of Al–Cu joints. The welding tool that was used in this study was made of AISI H-13 tool steel with a shoulder that was 15 mm in diameter and a stirring probe with a four-mm diameter and two-mm depth, as shown in Figure 1b. Pure Cu and brass plates were always positioned at the advancing side (AS). The tool was in the center of the Al–Cu joints, and it was not shifted to the Al or Cu side. According to our preliminary trial experiments of several Al–Cu joints and the macrograph/micrograph inspections for the welds of various parameters, optimum welding parameters were obtained for the welds with an optimal tensile strength and defect-free microstructures in the welding zone at the stable part of the seam for the present study. Based on our preliminary trials and the literature review [22], a low transverse speed and high tool rotational speed are usually required for obtaining defect-free joints. The tool rotational speed was set at 3000 rpm. The rotating tool was tilted 1.5° opposite to the welding direction, and the stirring probe moved along the butt line of the Al–Cu joint specimens at a constant traverse speed of about 60 mm min^−1^. The downward push pressure was controlled at about 45 MPa. During the FSW process, the downward push pressure was maintained for an appropriate time to generate sufficient frictional heat. The frictional heat softened the Al–Cu joint specimens, and the stirring probe caused material plastic flow in both circumferential and axial directions. The welded direction (WD), normal direction (ND), and transverse direction (TD) of the FSW Al–Cu dissimilar joints were also defined in Figure 1a. After the friction stir welding process, the upper and lower surfaces of welded specimens were carefully ground with 2000-grit SiC abrasive paper to eliminate the defects and stress concentrators located on the surface of the seam.

The FSW joined A1/C1, A6/C1, and A1/C2 dissimilar joints were cross-sectioned along the TD. For the study of the welding microstructures, the cross-sections (on the WD plane) taken at the stable part of the seam were ground and polished with a diamond polishing agent. The specimens were etched in a Keller’s solution for the Al alloy side, and the Cu alloy side was etched with a solution of five grams of FeCl_3_, 50 mL of HCl, and 100 mL of H_2_O. The phase composition within the welding zone (WZ) of the Al–Cu dissimilar joints were identified by an X-ray diffractometer (XRD, Bruker D8A25, Bruker Corp., Karlsruhe, Germany), using CuKα radiation at 40 kV and 40 mA with a scan speed of 3° (2θ) min^−1^ (step size, 0.02°). Microstructures of the A1/C1, A6/C1, and A1/C2 dissimilar joints were observed by the backscattering electron image (BEI) taken with a scanning electron microscopy (SEM, JEOL/JSM-6360, JEOL Ltd., Tokyo, Japan). SEM equipped with an energy-dispersive X-ray spectroscopy (EDS) and electron probe micro-analyzer (EPMA, JEOL JXA-8530F, JEOL Ltd., Tokyo, Japan) were used to identify the elemental compositions and distribution of compounds formed within the WZ of FSW Al–Cu dissimilar joints for the investigation of metallurgical interactions.

The Micro-Vickers hardness test (HV) was applied to evaluate the variations of microhardness after the FSW process. The Micro-Vickers hardness test across the cross-sections of the A1/C1, A6/C1, and A1/C2 joints was applied using a Vickers indenter (Future-Tech Corp., Kawasaki, Japan) with a 50-g load for 10 s of dwell time. Each measured microhardness datum was the average of at least three tests.The tensile strength of the FSW A1/C1, A6/C1, and A1/C2 dissimilar joints was measured according to the standard tension testing of ASTM E8M-11. Uniaxial tensile tests of the FSW Al–Cu joints were carried out in the directions perpendicular to the WD. Figure 1c shows the dimensions of the tensile specimens prepared from welded samples. The specimens were tested at room temperature with an initial strain rate of 8 × 10^−4^ s^−1^ per mm. The tensile strength measurements of each condition (A1/C1, A6/C1, and A1/C2 joints) were performed on 20 test specimens (*n* = 20) for the statistical significance of following Weibull statistical analysis. The fracture surfaces and sub-surfaces of the FSW Al–Cu joints were further examined using SEM/BEI with EDS mapping to analyze the fracture morphologies and behaviors.

## 3. Results

### 3.1. Microstructures and Microhardness Variation of FSW Al–Cu Dissimilar Joints

**Figure 2:**
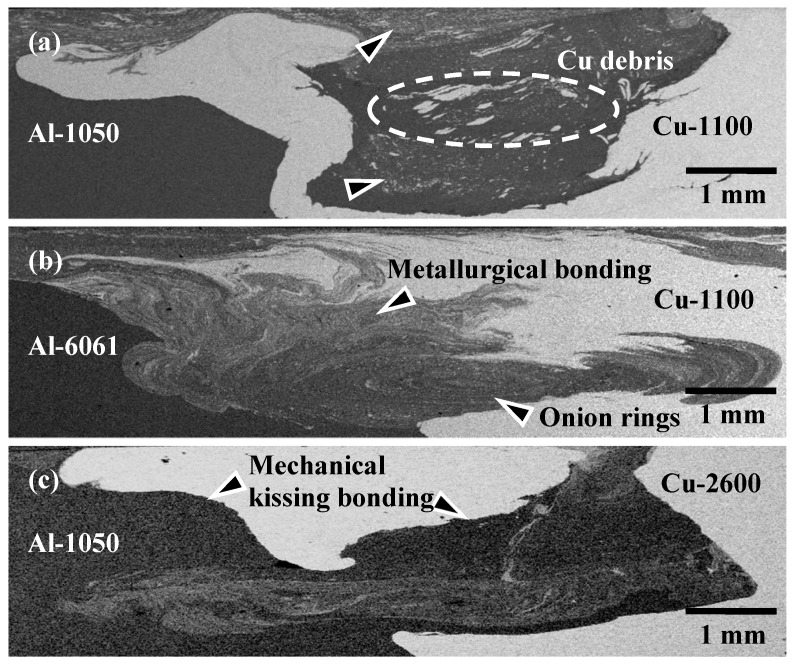
Scanning electron microscopy (SEM)/backscattering electron image (BEI) cross-sectional micrographs (on the welded direction (WD) plane) of the FSW (**a**) A1/C1, (**b**) A6/C1, and (**c**) A1/C2 joints (both of the C1 and C2 base metals were positioned at the advancing side).

Figure 2 represents the backscattering electron images (SEM/BEI) of cross-sectional micrographs within the WZ of various FSW Al–Cu dissimilar joints for illustration. The cross-sectional images are taken at the stable part of the seam. The light gray area is the Cu matrix, and the dark gray area is the Al matrix. It can be seen that sound A1/C1, A6/C1, and A1/C2 dissimilar joints are successfully achieved by the FSW process, while the pure Cu (C1100) and brass (C2600) plates are always positioned at the advancing side (AS). The good welding quality of all of the Al–Cu joints is obtained in the present study without the typical cavity defects that tend to occur within the WZ region.

Comparing Figure 2a with Figure 2b, it is apparent that the plastic flow of the Al and Cu base metals within the WZ region is quite different between the A1/C1 and A6/C1 joints. Figure 2a shows the cross-sectional microstructure of the A1/C1 joint for illustration. A large amount of pure Cu matrix and several dispersed large irregular Cu debris (as those encircled in Figure 2a) are mixed with the Al matrix in the WZ of the A1/C1 joint. A clear A1/C1 joint boundary represents that just a mechanical kissing bond is formed between AA1050H-aluminum and C1100-pure Cu at the welding interface of A1/C1 specimens after the FSW process. In addition, a small amount of Al–Cu reacting mixture, which is the result of the intense material plastic flow during FSW, is observed at the top and near the bottom of the WZ (indicated by the triangular marks). The mixture can be considered the metallurgical bonded zone of AA1050H-aluminum and C1100-pure Cu base metals. Unlike the A1/C1 joint, no typical dispersed Cu debris or small Cu particles are observed within the WZ of the A6/C1 joint, as shown in Figure 2b. It is worth noting that a significant wide range of material plastic flow is observed between the Al and Cu matrixes with an obvious onion rings microstructure (indicated by the triangular marks) in the WZ of the A6/C1 joint. It can be recognized that the A6/C1 joint displays much better welding quality, with a larger area fraction of its metallurgical bonded zone and no mechanical kissing bond between the AA6061-T6-aluminum and C1100-pure Cu base metals, compared with the A1/C1 joint. Figure 2c shows the cross-sectional microstructure of the A1/C2 joint for illustration. Comparing Figure 2c with Figure 2b, an Al–Cu metallurgical bonded zone, which is the result of the material plastic flow of AA1050H-aluminum and C2600-brass during FSW, is formed within the WZ of the A1/C2 joint (Figure 2c). However, the Al–Cu metallurgical bonding area fraction of the A1/C2 joint is apparently less than that of the A6/C1 joint. Moreover, a clear joint boundary between AA1050H-aluminum and C2600-brass base metals is observed in the WZ region. Unlike the metallurgical bonding effect within the Al–Cu reacting mixture during FSW, the clear joint boundary observed in the A1/C2 jointis thought of as a mechanical kissing bonded interface, as indicated by the triangular marks in Figure 2c.

**Figure 3:**
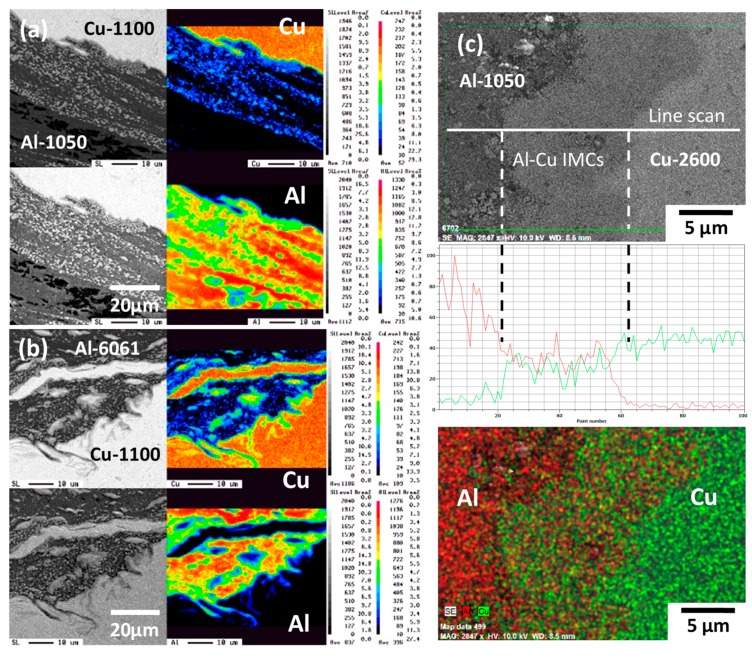
The electron probe micro-analyzer (EPMA) analysis for Al and Cu elements distribution within the WZ of (**a**) the FSW A1/C1 joint, and (**b**) the FSW A6/C1 joint. (**c**) The SEM/BEI line-scanning and Al–Cu elements mapping at the Al/Cu interface of the FSW A1/C2 joint.

Figure 3a,b show the SEM/BEI images and EPMA analysis results for the element distribution maps of Al and Cu within the WZ of the A1/C1 and A6/C1 joints, respectively. As shown in Figure 3a, an apparent joint boundary exists at the welding interface of the AA1050H-aluminum (the dark gray region) and C1100-pure Cu (the light gray region) base metals. In addition, some bright small particles, which are mainly composed of the Cu element, are detected within the AA1050H-aluminum matrix (see Figure 3a). These regions can be recognized as the reacting mixtures resulted from the intense material plastic flow of Al and Cu base metals during FSW, as indicated by the triangular marks in Figure 2a. It can be reasonably deduced that some Al–Cu intermetallic compounds (IMCs) are formed within the Al–Cu reacting mixture region, and the phase composition of Al–Cu IMCs will be identified by following X-ray diffraction analysis. Figure 3b shows the SEM/BEI images and EPMA analysis results in the WZ of the A6/C1 joint. The dark gray and light gray regions correspond to the AA6061-T6-aluminum and C1100-pure Cu base metals, respectively. As shown in Figure 3b, the FSW A6/C1 joint displays a significant material plastic flow and reacting mixtures within the WZ compared with the A1/C1 joint. Therefore, comparing Figure 3b with Figure 2b demonstrates that the metallurgical bonded zone consists of severe stirring plastic flow between AA6061-T6-aluminum and C1100-pure Cu base metals with a large amount of reacting mixtures of dispersed Al–Cu IMCs particles. The distribution of Al–Cu IMCs particles and microstructural morphologies of the A6/C1 joint are quite different from that of the A1/C1 joint. Figure 3c shows the SEM/BEI image and a line scanning analysis result with the distribution of Al and Cu elements in the WZ of the FSW A1/C2 joint. The dwell time via the SEM/BEI EDS mapping to collect the data for the Al and Cu elements is about 10 minutes. The dark gray and light gray regions correspond to the AA1050H-aluminum and C2600-brass base metals, respectively. However, unlike the reacting mixtures represented in Figure 3a,b, an obvious interfacial layer with mixing Al and Cu elements is observed between the base metals, and it can be recognized as the Al–Cu IMCs thick layer that was formed on the interface of AA1050H-aluminum and C2600-brass during the FSW process. Generally, the FSW dissimilar joints are failed at the WZ or along the interface between the base metals after the mechanical tests. Therefore, the tensile strength, measuring data fluctuation, failure behaviors, and welding reliability of the FSW A1/C1, A6/C1, and A1/C2 joints will be strongly affected by the metallurgical bonding effect, the particle distribution, and the morphologies of reacted Al–Cu IMCs within the WZ. As a result of the above-mentioned observation and analysis of microstructures, the correlation between the microstructural features, tensile strength, and reliability of these FSW Al–Cu dissimilar joints will be discussed in the following sections.

**Figure 4:**
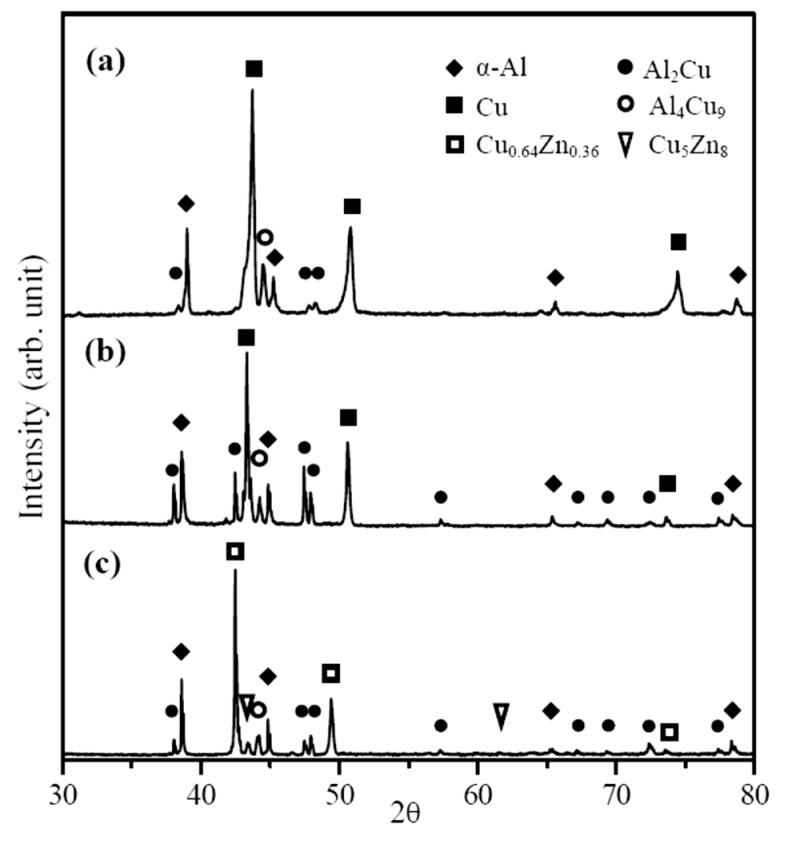
X-ray diffraction patterns of the friction stir welded (**a**) A1/C1, (**b**) A6/C1, and (**c**) A1/C2 dissimilar joints within the welding zone (WZ).

The X-ray diffraction patterns obtained from the WZ region of various FSW dissimilar joints are given in Figure 4. Figure 4a,b show the XRD patterns of the A1/C1 and A6/C1 joints, respectively. In addition to the strong diffraction peaks of major α-Al (main peaks detected at 2θ = 38.47°, 44.74°, 65.13°, and 78.23°, JCPDS 04-0787) and Cu (main peaks detected at 2θ = 43.30°, 50.43°, and 74.13°, JCPDS 04-0836) base metals, some relatively weak peaks are also detected within the WZ region of these Al–Cu dissimilar joints. According to the Al–Cu equilibrium phase diagram, several Al–Cu IMCs, such as AlCu (η_2_), Al_2_Cu (θ), Al_2_Cu_3_ (ε), Al_3_Cu_4_ (ζ_2_), and Al_4_Cu_9_ (γ) can be found in the Al–Cu binary alloy system. However, it is recognized that the Al-rich Al_2_Cu phase and Cu-rich Al_4_Cu_9_ phase are the two major IMCs formed during the Al–Cu metallurgical reaction [23,26,41]. Therefore, these weak diffraction peaks obtained in the WZ region of A1/C1 joints are then identified as the IMCs of Al_2_Cu (main peaks detected at 2θ = 37.87°, 42.59°, 47.33°, and 47.81°, JCPDS 25-0012) and Al_4_Cu_9_ (the diffraction peak detected at 2θ = 44.12°, JCPDS 24-0003). Comparing Figure 4b with Figure 4a, we can see that the diffraction peaks of the Al_2_Cu and Al_4_Cu_9_ IMCs for the A6/C1 joint are significantly sharper and much more obvious than those of the A1/C1 joint. The differencein peak intensity means that more Al_2_Cu and Al_4_Cu_9_ IMCs are obtained within the WZ region of the A6/C1 joint. Referring to the SEM/BEI images and the element distribution maps obtained by EPMA, the XRD analysis result of the FSW A6/C1 joint (see Figure 4b cf. Figure 2b and Figure 3b) corresponds to the microstructural feature, which displays a more widely Al–Cu reacting mixture distribution with a larger area fraction of the metallurgical bonded zone than the FSW A1/C1 joint (see Figure 4a cf. Figure 2a and Figure 3a). Figure 4c is the XRD pattern of the FSW A1/C2 dissimilar joint, which included strong diffraction peaks of α-Al and Cu_0.64_Zn_0.36_ (α-brass, main peaks detected at 2θ = 42.33°, 49.28°, and 72.25°, JCPDS 50-1333) base metals. The result illustrates that the WZ region of the A1/C2 joint also consists mainly of Al and Cu (i.e., α-brass) base metals with a fair amount of Al_2_Cu and Al_4_Cu_9_ IMCs. In addition, a γ-Cu_5_Zn_8_ compound (diffraction peaks at 2θ = 43.30° and 62.88°, JCPDS25-1228) is also observed in the A1/C2 joint, as indicated by the triangular marks in Figure 4c. As a result, it can be recognized that the γ-Cu_5_Zn_8_ compound is another minor reaction product accompanied by the formation of Al_2_Cu and Al_4_Cu_9_ IMCs for the A1/C2 joint during FSW. Since less Al–Cu reacting mixture and a clear mechanical kissing bonded boundary of the A1/C2 joint are observed (see Figure 2c), it implies that the weldability of A1/C2 joint is worse than that of the A1/C1 and A6/C1 joints.

**Figure 5:**
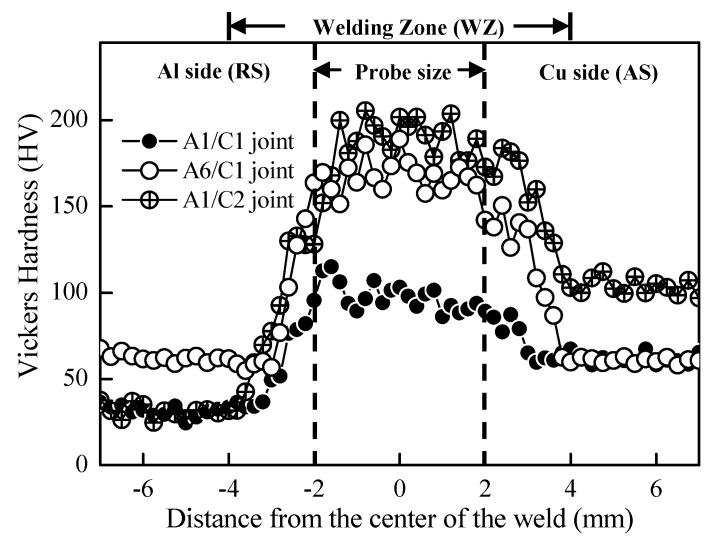
The variation of microhardness of various FSW Al–Cu dissimilar joints. The indentations are made with a spacing of 0.2 mm on the WD plane (AS: advancing side; RS: retreating side).

Figure 5 displays the distribution of micro-Vickers hardness (HV) profiles measured within the WZ regions of FSW A1/C1, A6/C1, and A1/C2 dissimilar joints. The hardness data recorded on the transverse cross-sections of FSW-joined specimens, and the Vickers indenter testing positions are located at 1.5 mm depth from the surface. The micro-Vickers hardness test reveals the average values of AA1050H, AA6061-T6 aluminum alloys, commercial pure copper (C1100), and brass (C2600) base metals to be about HV32.6 ± 4.7, HV61.2 ± 5.3, HV62.1 ± 3.0, and HV103.2 ± 4.5, respectively. According to the profiles represented in Figure 5, the hardness of FSW Al–Cu dissimilar joints significantly increases in the WZ region relative to both Al and Cu base metals. The data fluctuation at the transverse direction is the resulted of the heterogeneous microstructure of the WZ. Referring to the cross-sectional microstructures as mentioned in Figure 2, the distribution range of the increased hardness profiles (in the position of about ± 4 mm from the center, as shown in Figure 5) is almost the same as that of the Al–Cu metallurgical bonded region with obvious metallic plastic flow. Therefore, it can be recognized that the increased hardness profiles of the A6/C1 and A1/C2 joints can be attributed to the apparent Al–Cu reacting mixture within the WZ region (see Figure 5 cf. Figure 2b,c). In addition, the high hardness values of the A6/C1 and A1/C2 joints are also the result of the significant strain-hardening effect of intense materials plastic deformation as well as the particle strengthening effect with the formation and uniform redistribution of Al_2_Cu, Al_4_Cu_9_, and a fair amount of γ-Cu_5_Zn_8_ IMCs (such as those observed by the XRD analysis in Figure 4) during the FSW process. However, compared with the A6/C1 and A1/C2 joints, the hardness profile for the A1/C1 joint is lower as a result of an insufficient metallurgical bonding reaction and a lesser amount of Al–Cu reacting IMCs mixture (see Figure 2a cf. Figure 4).

### 3.2. Tensile Failure Strength of FSW Al–Cu Dissimilar Joints

**Figure 6:**
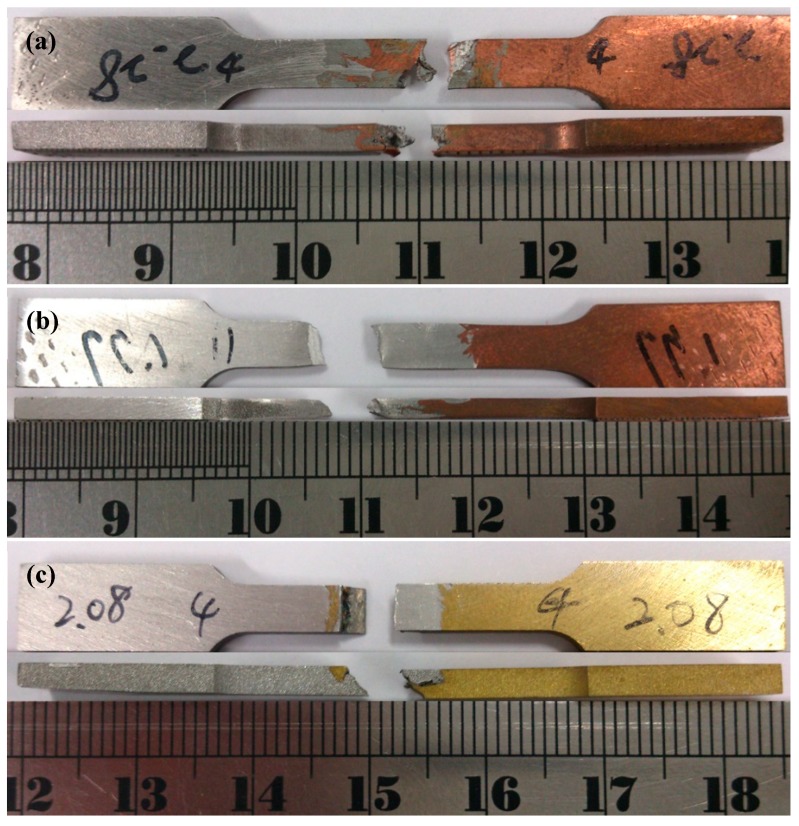
Macrographs of fractured FSW (**a**) A1/C1, (**b**) A6/C1, and (**c**) A1/C2 dissimilar joints after tensile tests.

The tensile strength of the FSW dissimilar Al–Cu joint is evaluated as a criterion to the joint performance. Table 2 lists a comparison of the tensile strength and elongation of base metals and the FSW A1/C1, A6/C1, and A1/C2 dissimilar joints. Since a sound FSW A6/C2 joint without welding cavity defects in the WZ region is not successfully obtained, therefore, the tensile properties of A6/C2 joints are too low to be used in this study. In addition, the data fluctuation of A6/C2 joints is dispersed to show a valid statistical significance for the following reliability analysis in this study. It can be seen that a maximum tensile strength of about 212.7 MPa and the highest elongation of about 22.3% are obtained for the FSW A6/C1 joint. Figure 6 shows the representative fractured specimens of FSW A1/C1, A6/C1, and A1/C2 dissimilar joints after tensile tests. The Al–Cu dissimilar joints failed with distinguished fracture characteristics, and the fracture location can be clearly observed in these photos. The fracture of FSW A1/C1 joints is usually located at the WZ region, as shown in Figure 6a. Since the average tensile strength of A1/C1 joints is about 85% of the AA1050H aluminum alloy (121.8 ± 2.3 MPa) according to earlier measurements for base metals, the fracture characteristics that are displayed in Figure 6a and the decrease in the tensile strength of the A1/C1 joints should be the result of the inhomogeneous welding microstructure, which features a mechanical kissing bond at the interface between AA1050H-aluminum and C1100-pure Cu (see Figure 2a cf. Figure 6a).

Comparing Figure 6b with Figure 6a, it is worth noting that the fracture region of the FSW A6/C1 joints is distant from the WZ and nearly located at the heat-affected zone (HAZ) with an obvious necking deformation of the AA6061-T6 Al base metal. As a result, both the tensile strength and the elongation of the A6/C1 joints are significantly higher than that of the A1/C1 joints (see Table 2), and the fracture characteristic without failure at the WZ of the FSW A6/C1 joints, which is represented in Figure 6b, can be regarded as an illustration of better joint quality. Referring to the SEM/BEI cross-sectional images and EPMA analysis results shown in Figure 2b and Figure 3b, it is noted that the much better tensile strength of the A6/C1 joints is achieved from their good joint quality with a larger area fraction of the metallurgical bonded zone and a firmly material metallurgical joining between the AA6061-T6 Al alloy and the C1100 commercial pure copper through the FSW process. The intense material plastic flow with obvious onion rings (see Figure 2b) and uniformly dispersive refined hard IMCs particles in the Al–Cu reacting mixture enhance the metallurgical bonding effect within the WZ. Meanwhile, the movement of dislocations will be effectively impeded by the tangle of high dislocation density and the refined hard IMCs particles during plastic deformation. Therefore, it can be recognized that the FSW A6/C1 dissimilar joint with increased hardness profiles and much higher tensile strength is obtained due to the strong metallurgical bonding effect.

Among these Al–Cu dissimilar joints, the FSW A1/C2 joint apparently exhibits the worst tensile strength and displays much lower elongation (see Table 2), with an obvious brittle fracture at the WZ region along the joining interface of AA1050H-aluminum and C2600-brass base metals, as illustrated in Figure 6c. The notable degradation in the tensile strength of the A1/C2 joints should be closely correlated with the structural inhomogeneity. Referring to Figure 2c and Figure 3c cf. Figure 6c, a large area fraction of the mechanical kissing bond with a continuous thick IMCs layer was significantly formed at the Al/brass interface within the WZ of the A1/C2 joints. It demonstrates that the reduction of effective metallurgical bonding and the formation of a continuous thick IMCs layer yielded a weak interfacial A1/C2 joint. As a result, the detrimental decohesive ruptures notably occurred at the weak mechanical kissing bonded boundary of AA1050H-aluminum and C2600-brass before other structural defects caused the failures of the A1/C2 joint throughout the WZ.

According to the above-mentioned examinations, FSW A1/C1, A6/C1, and A1/C2 dissimilar joints display quite different welding microstructures, interfacial joining characteristics, hardness profiles, and phase compositions within the WZ regions. Since the tensile strength of FSW Al–Cu dissimilar joints should be closely affected by the variation of welding qualities, the statistical analysis by the Weibull model on the data variance of tensile tests made it possible to assess the microstructural features in determining the failure behaviors and reliability of FSW Al–Cu dissimilar joints. The Weibull statistical analysis detail on the tensile mechanical properties and failures related to the welding qualities of FSW Al-Cu joints will be discussed in the following section.

## 4. Discussion

### 4.1. The Weibull Statistical Analysis on the Failure Probability of Al–Cu Joints

The failure of an engineering component, as defined by reliability engineering, can be recognized as “the event, or inoperable state, in which any item or part of an item does not, or would not, perform as previously specified” [42]. The advantage of Weibull statistical analysis for reliability engineering is to effectively evaluate failure probability and provide reasonable failure predictions for engineering components from the stages of design and manufacturing processes with extremely small amount of testing samples. In this study, the cumulative failure probability function (Equation (1)) determined by the three-parameter Weibull model is applied to simulate the data variability of the measured tensile strength of FSW Al–Cu dissimilar joints. The cumulative failure probability *F*(*σ_i_*) of the testing samples is estimated using Bernard’s median rank [43], and the reliability function *R*(*σ_i_*) with a relation of *R*(*σ_i_*) = 1 − *F*(*σ_i_*) is determined as the survival probability. The cumulative failure probability is controlled by parameters *m*, *σ_c_*, and *σ*_0_. The Weibull modulus (*m*) is a measure of data variability. The characteristic life (*σ_c_*) corresponds to the tensile strength at which the cumulative failure probability is equal to 63.2%. The minimum strength (*σ*_0_) is so-called the failure-free strength, which means that the failure probability of the FSW Al–Cu dissimilar joints below this tensile strength is zero.

(1) $$F(\sigma_{i})=\int_{\sigma =0}^{\sigma =\sigma_{i}}f\left(\sigma \right)d\sigma =1-\exp\left[-{\left(\frac{\sigma_{i}-\sigma_{0}}{\sigma_{c}}\right)}^{m}\right]$$

**Figure 7:**
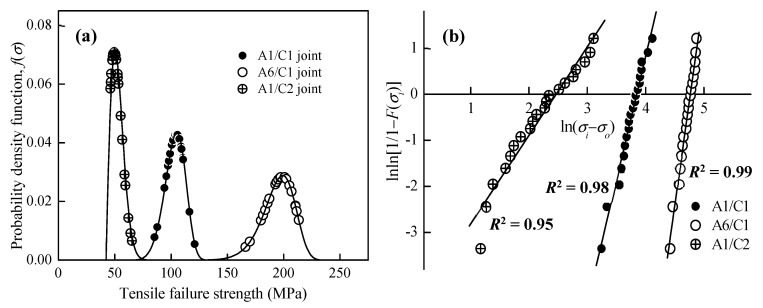
(**a**) The failure probability density function *f*(*σ_i_*) curves, and (**b**) the Weibull distribution plots of various FSW Al–Cu dissimilar joints. *F*(*σ_i_*) is the cumulative failure probability for a corresponding tensile strength (*σ_i_*), and the slope represents the Weibull modulus (*m*) calculated by the least squares fitting method at a maximum coefficient of determination (*R*^2^).

Fitting the measured tensile strength data into the cumulative failure probability function of Equation (1), and subsequently the failure probability density function (*f*(*σ_i_*)) of FSW A1/C1, A6/C1, and A1/C2 dissimilar joints are calculated and plotted in Figure 7a. Figure 7b illustrates the Weibull plot, which is a natural logarithmic (ln) graph for the cumulative failure probability at each corresponding *σ_i_* of various FSW Al–Cu dissimilar joints. The horizontal scale of the Weibull plot is a measure of tensile strength, and the vertical scale is the cumulative percentage failed. The Weibull modulus, which is particularly significant and provides a clue to the physics of the failure, is graphically evaluated from the slope of Weibull plots by the least squares fitting method at a maximum coefficient of determination (*R*^2^). According to the definition of the Weibull model, the critical coefficient of determination (CCD) for 20 failures (*n* = 20) should be higher than 0.95, and then the Weibull plot can be considered a good fit for the three-parameter Weibull cumulative distribution function [44]. Therefore, it is recognized that a good linear relationship is obtained for the experimental data in Figure 7b, and the Weibull model is valid to describe the failure behaviors of the FSW Al–Cu joints. The Weibull statistical analysis results are listed in Table 3.

### 4.2. Microstructural Variations Affect Data Fluctuation and Failure Behaviors

**Figure 8:**
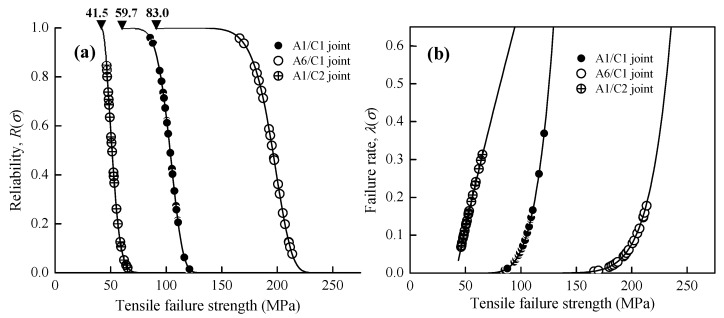
(**a**) The reliability function curves *R*(*σ_i_*) and (**b**) the failure rate curves from the hazard function *λ*(*σ_i_*) of various FSW Al–Cu dissimilar joints. These curves start from the minimum strength (*σ*_0_), which is the safety tensile strength for FSW Al–Cu dissimilar joints.

Referring to the aforementioned microstructural observations and tensile testing results, a fair amount of the data fluctuation for the FSWA1/C1, A6/C1, and A1/C2 dissimilar joints shown in Figure 7a should be significantly related to the joint inhomogeneity with a different metallurgical bonding area fractionin the WZ region. Since the Weibull model is commonly adopted to forecast the reliability and failure behaviors of engineering components, the hazard function (*λ*(*σ_i_*)) listed in Equation (2) at each corresponding tensile strength is further defined as the ratio of the failure probability density function and reliability functionin the present study for the assessment of failure behaviors. Figure 8a shows the reliability function (*R*(*σ_i_*)) curves of the Al–Cu joints. These curves start from the minimum strength (*σ*_0_), and the reliability of joints is decreased with increasing tensile loading. The failure rate curves calculated from the hazard function (*λ*(*σ_i_*)) of Al–Cu joints are shown in Figure 8b.

(2) $$\lambda \left(\sigma_{i}\right)=\frac{f\left(\sigma_{i}\right)}{R\left(\sigma_{i}\right)}=\frac{m}{\sigma_{c}{}^{m}}{\left(\sigma_{i}-\sigma_{0}\right)}^{m-1}$$

The Weibull modulus (*m*) is a dimensionless value, and it is a main factor for the Weibull model to determine which Al–Cu joint displays better engineering reliability. It is noted that the Weibull modulus represents the variability of experimental data, which becomes larger as the degree of tensile strength fluctuation decreases and the reliability of the joints increases. As a result of Table 3 and Figure 8b, the Weibull statistical analysis demonstrates that all of the A1/C1, A6/C1, and A1/C2 welding conditions are reliable FSW dissimilar joints with a wear-out failure model (*m* > 1) of the increasing failure rate (IFR) behavior. However, the A1/C1 joint (*m* = 5.4) and the A6/C1 joint (*m* = 9.2) significantly show a larger Weibull modulus compared with the A1/C2 joint (*m* = 1.7). The A1/C2 joints with a lower Weibull modulus generally characterize an early failure behavior because of the much higher initial failure probability of the tensile specimens compared with the A1/C1 and A6/C1 joints, as shown in Figure 7a. Therefore, referring to the microstructural observations illustrated in Figure 2 and Figure 3, it is demonstrated that the FSW Al-Cu dissimilar joints with a large amount of Al–Cu reacting mixtures of uniformly dispersed Al–Cu IMCs particles can resist a higher tensile failure load and also display better engineering reliability because of the larger Weibull modulus. The FSW A6/C1 dissimilar joint with a successful metallurgical bonded WZ region (see Figure 2a and Figure 3a cf. Figure 6b) represent better tensile strength and reliability than a mainly mechanical kissing bonded A1/C2 joint interface (see Figure 2c cf. Figure 6c). Moreover, the reliability of FSW A1/C2 joints is rapidly decreased through just slightly increasing the tensile loading to be larger than the minimum strength, as shown in Figure 8a. Since the minimum strength can be recognized as the safety value of an engineering component, the existence of minimum strength *σ*_0_ is needed in order to evaluate the critical reliable tensile strength of varied FSW Al–Cu dissimilar joints. Therefore, FSW Al–Cu dissimilar joints with a larger Weibull modulus can properly be selected for engineering application, as this may be an indicator of lower technique sensitivity and less reliability decrease (see Figure 8a) while the applied tensile loading exceeds the minimum strength.

**Figure 9:**
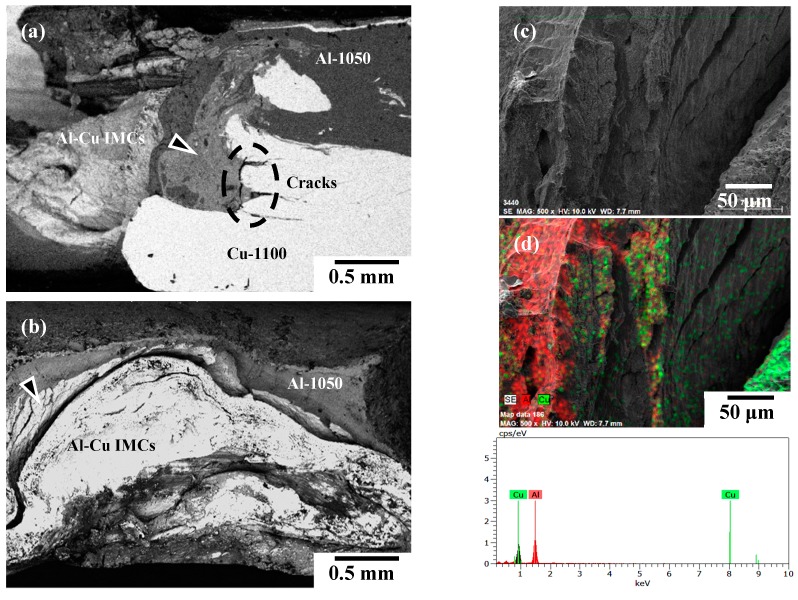
(**a**) Failure sub-surface, (**b**) fracture surface, (**c**) representative fracture morphology denoted by the arrow in (**b**), and (**d**) Al, Cu elements energy-dispersive X-ray spectroscopy (EDS) mapping of the A1/C1 joint.

**Figure 10:**
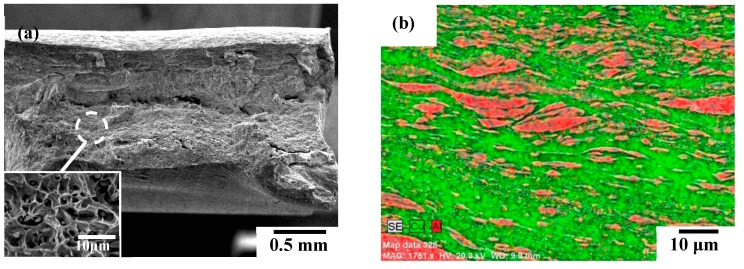
(**a**) Fracture surface and (**b**) EDS mapping of the Al and Cu elements at the WZ region of the tensile failed A6/C1 joint.

**Figure 11:**
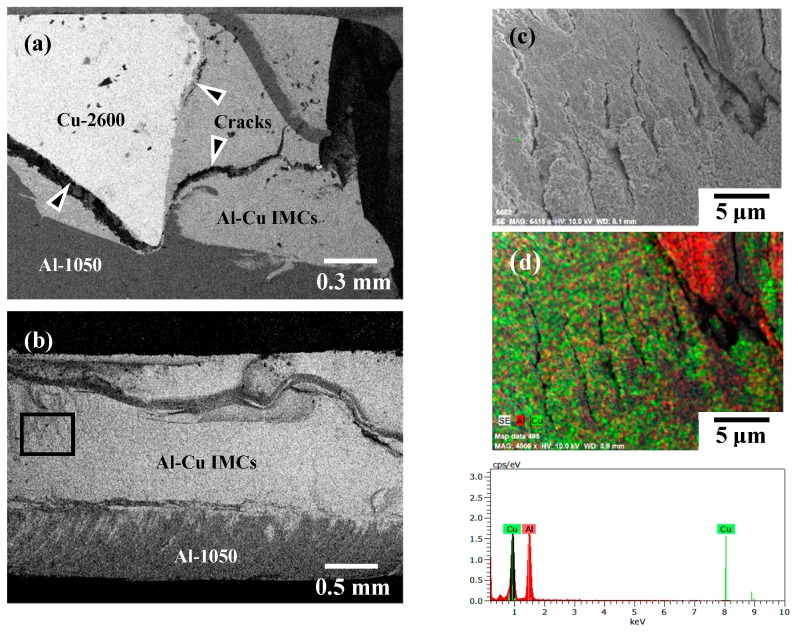
(**a**) Failure sub-surface, (**b**) fracture surface, (**c**) representative fracture morphology of the rectangular area in (**b**), and (**d**) EDS mapping of the Al and Cu elements of the A1/C2 joint.

Properly friction stir welded joints should have high tensile strength and display ductility, whereas the joints with low tensile strength will fail in a brittle fracture at the WZ region. In order to realize the fracture mechanism with the above-mentioned Weibull statistical analysis results, Figure 9, Figure 10 and Figure 11 show the representative SEM fracture morphologies of the FSW A1/C1, A6/C1, and A1/C2 dissimilar joints, respectively. Figure 9a gives the BEI fracture sub-surface of A1/C1 joints to identify the Al and Cu base metals and Al–Cu IMCs more clearly. A fair amount of reacting mixtures (the light gray region indicated by the triangular mark) composed of Al_2_Cu and Al_4_Cu_9_ IMCs (identified by XRD analysis, see Figure 4a) are observed within the WZ region of the A1/C1 joint. In addition, cracks propagation is observed at the IMCs/Cu interface, and the internal cracks are perpendicular to the tensile direction, as those encircled in Figure 9a. Figure 9b shows the BEI tensile fracture surface of the A1/C1 joints. Figure 9 c,d represent the EDS mapping analysis region denoted by the triangular mark in Figure 9b, and the composition of this region is about 54.8 Al and 45.2 Cu (in atomic %) by semi-quantitative SEM/EDS analysis. It can be seen that a brittle fracture appears, and the fracture can be recognized as a result of the interfacial cracks propagation along the Al–Cu IMCs within the WZ region (see Figure 9b cf. Figure 2a and Figure 6a).

Figure 10a shows the SEM tensile fracture surface of the A6/C1 joints for illustration. As seen from the fracture morphologies of the A6/C1 joint, a ductile failure behavior appears with necking and dimpled ruptures (see Figure 10a cf. Figure 6b) at the AA6061 Al base metal. Therefore, it is recognized that A6/C1 joints obviously display better elongation (as listed in Table 2) than A1/C1 and A1/C2 joints. Figure 10b displays the EDS mapping analysis results of Al and Cu elements (with chemical compositions of 31.2 Al and 68.8 Cu, in atomic %) obtained from the WZ region of tensile failed A6/C1 joints. Referring to the above-mentioned microstructural observations, significant reacting mixtures of dispersed Al–Cu IMCs particles represent that intense material plastic flow effectively induces a larger area fraction of the metallurgical bonded zone (see Figure 2b and Figure 3b cf. Figure 4b) within the WZ region of the A6/C1 joint during the FSW process. As a result, Figure 10b illustrates a typical WZ region of good welding quality with a textureof elongated joining materials flow along the tensile direction without an apparent cracking effect after tensile tests. Since the Al–Cu IMCs can be used as reinforcing phases through the distribution of particles [27,45], as shown in the experimental results mentioned in Table 2, Figure 8a and Figure 10b demonstrate that a large fraction of metallurgical bonding of obvious onion rings with dispersed Al–Cu IMCs particles provides a successful firm welding structure, higher tensile strength, better welding quality, and joint reliability of the FSW A6/C1 joint compared with the A1/C1 joint.

Figure 11a,b shows the BEI fracture sub-surface and fracture surface of the A1/C2 joints, respectively. Figure 11c,d represent the EDS mapping analysis result of the rectangular region denoted in Figure 11b, and the composition in this region is about 51.7 Al and 48.3 Cu (in atomic %) by SEM/EDS analysis. Referring to Figure 6c, the fracture of the A1/C2 joints is almost located at the A1050H-aluminum and C2600-brass joint interface. It is reported that FSW dissimilar joints with an excessively generated thick interfacial IMCs layer generally display poor mechanical properties because of the brittleness of IMCs and quite easier crack propagation [27,32]. Therefore, comparing Figure 11a with Figure 6c, it can be found that many of the cracks that were significantly initiated at the AA1050H/C2600brass joint interface (cracks are also observed on the fracture surface of Figure 11c), and the failure of joints occurred while the cracks penetrated through Al–Cu IMCs (as denoted by the triangular marks in Figure 11a). This phenomenon demonstrates that a thick continuous interfacial IMC results in a significant deterioration of tensile strength and the presence of much lower Weibull modulus (*m* = 1.7) with a rapid increase in the failure probability (see Table 3 and Figure 7a) for the A1/C2 joint. Therefore, FSW A1/C2 joints obviously display the lowest tensile strength with a brittle fracture and an unfavorable welding reliability in this study (see Figure 11a,b cf. Figure 8a).

## 5. Conclusions

The welding qualitiesand tensile mechanical properties related to the metallurgical bonded microstructural characteristicsof FSW Al–Cu dissimilar joints are identified and discussed in this study. In addition, the relationship between welding quality and tensile strength, as well as the joint reliability, is investigated by the Weibull statistical analysis. The conclusions are drawn based on the above results and discussions:

(1) Dissimilar Al–Cu joints of AA1050H/C1100-Cu (A1/C1), AA6061-T6/C1100-Cu (A6/C1), and AA1050H/C2600-brass (A1/C2) couples are successfully joined without typical cavity defects in the welding zone (WZ) by the friction stir welding process (FSW).
(2) Al_2_Cu and Al_4_Cu_9_ are the major intermetallic compounds (IMCs) formed in the metallurgical bonded welding zone of FSW Al–Cu dissimilar joints, and γ-Cu_5_Zn_8_ is another reacted IMC observed in the WZ of the AA1050H/C2600-brass joint.
(3) The microhardness of FSW Al–Cu joints in the WZ is increased as a result of the formation of Al–Cu IMCs and intense plastic deformation during FSW.
(4) The AA6061-T6/C1100-Cu joint exhibits a significant metallurgical bonded zone with onion rings in the WZ region, whereas the AA1050H/C2600-brass joint usually displays a mechanical kissing bonded boundary at the Al–Cu joining interface.
(5) Through the powerful statistical analysis of the Weibull model, FSW Al–Cu dissimilar joints, which display a wear-out failure model, can be recognized as reliable joints for further engineering applications.
(6) Better welding reliability and a higher tensile strength with ductile dimpled ruptures can be obtained for those FSW Al–Cu joints with IMCs particles uniformly dispersed in a large area fraction of the metallurgical bonded WZ region.
(7) FSW Al–Cu joints with a mechanical kissing bonded boundary and a thick continuous interfacial IMC layer results in a rapid increase in the failure probability and the deterioration of tensile strength with a brittle fracture at the WZ region of the joints.

## Figures and Tables

**Table 1:** Chemical compositions of the used Al and Cu base metals (in wt.%).

**Cu Base Metals**	**Cu**		**Zn**		**Pb**		**Fe**		**Si**	**Mg**	**Al**
C1100 (Pure Cu)	Bal.		0.03		0.02		0.01		0.01	-	0.02
C2600 (brass)	69.3		Bal.		0.01		0.03		0.15	0.03	0.02
**Al Base Metals**	**Al**	**Mg**		**Si**	**Fe**	**Mn**		**Cr**	**Cu**	**Zn**	**Ti**
AA1050H	Bal.	0.05		0.15	0.38	0.05		-	0.05	0.01	0.02
AA6061-T6	Bal.	1.03		0.66	0.35	0.11		0.14	0.22	0.03	0.02

**Table 2:** Tensile properties of base metals and various FSW Al–Cu dissimilar joints.

Samples	TensileStrength (MPa)	Elongation (%)
AA1050H *	121.8 ± 2.3	15.2±1.7
AA6061-T6 *	293.1 ± 2.6	12.8± 2.5
C1100 Cu *	227.9 ± 1.8	30.6 ± 1.8
C2600 Brass *	365.2 ± 1.5	27.7 ± 2.3
A1/C1 Joint ^†^	108.6 ± 9.1	10.5 ± 3.3
A6/C1 Joint ^†^	212.7 ± 8.5	22.3 ± 2.8
A1/C2 Joint ^†^	53.2 ± 5.9	5.7 ± 0.9

* Each value was the average of a least three tests; ^†^ Each value was the average of 20 tests (*n* = 20).

**Table 3:** Statistical analysis results * of the Weibull model for the tensile strength of various FSW A1–Cu dissimilar joints.

Samples	Weibull Modulus, *m*	Characteristics Strength (MPa), *σ_c_*	Minimum Strength (MPa), *σ*_0_	Coefficient of Determination, *R*^2^
A1/C1 Joint	5.4	46.9	59.7	0.98
A6/C1 Joint	9.2	117.5	83.0	0.99
A1/C2 Joint	1.7	11.7	41.5	0.95

* Data were calculated from Weibull plots (Figure 7b).
